# Monkeypox in a patient with undifferentiated connective tissue disease

**DOI:** 10.1093/rheumatology/keac568

**Published:** 2022-10-04

**Authors:** Tommaso Clemente, Diana Canetti, Lorenzo Dagna, Caterina Uberti Foppa, Antonella Castagna, Vincenzo Spagnuolo, Silvia Nozza, Corrado Campochiaro

**Affiliations:** Vita-Salute San Raffaele University, Milan, Italy; Infectious Diseases, IRCCS San Raffaele Scientific Institute, Milan, Italy; Infectious Diseases, IRCCS San Raffaele Scientific Institute, Milan, Italy; Vita-Salute San Raffaele University, Milan, Italy; Unit of Immunology, Rheumatology, Allergy and Rare Diseases, IRCCS San Raffaele Scientific Institute, Milan, Italy; Vita-Salute San Raffaele University, Milan, Italy; Infectious Diseases, IRCCS San Raffaele Scientific Institute, Milan, Italy; Vita-Salute San Raffaele University, Milan, Italy; Infectious Diseases, IRCCS San Raffaele Scientific Institute, Milan, Italy; Infectious Diseases, IRCCS San Raffaele Scientific Institute, Milan, Italy; Infectious Diseases, IRCCS San Raffaele Scientific Institute, Milan, Italy; Vita-Salute San Raffaele University, Milan, Italy; Unit of Immunology, Rheumatology, Allergy and Rare Diseases, IRCCS San Raffaele Scientific Institute, Milan, Italy

Rheumatology key message

- Currently, rheumatologists should always rule out monkeypox as the cause of new-onset skin rashes.

Dear Editor, Since May 2022, a global outbreak of monkeypox (MPX) has been ongoing [1]. MPX virus (MPXV) is a zoonotic double-stranded DNA orthopoxvirus, related to variola virus. It is primarily endemic in West and Central Africa, although outbreaks outside this region have been described [2]. Human-to-human transmission (through skin lesions, droplets or fomites) is generally incidental [3]. Nonetheless, in the current MPX spread, some clues, such as the identification of viral DNA from seminal fluid, the atypical localization of lesions on genital and peri-anal regions, and the primarily diffusion in men who have sex with men point towards a possible sexual transmission [4]. Clinical presentation is smallpox-like, but less severe [5]. On 23 July 2022 the World Health Organization defined this MPX outbreak as an international public health emergency, in light of the number of cases (>16 000 in 75 countries) and its rapid increase [6]. In Italy, 599 infections have been detected to date [7].

To the best of our knowledge, no data on MPX in rheumatic patients on immunosuppressive therapy have been published yet. Herein, we report a case of MPX in an individual with UCTD.

In June 2022, a 66-year-old bisexual man presented to our sexually transmitted disease clinic at San Raffaele Scientific Institute, Milan, Italy, for the onset of pustular lesions on his left hand, penis and scrotum, which had progressively worsened over 10 days (Fig. 1). He had no fever or lymphadenopathies. His medical history was significant for hypertension and UCTD. The patient had been diagnosed with UCTD in 2019, due to the presence of Raynaud’s phenomenon, photosensitivity rash, inflammatory arthritis of wrists and proximal interphalangeal joints, and positivity for ANA 1:640 with granular pattern and anti-Ro52. He had been started on hydroxychloroquine 200 mg twice daily and prednisone which had been tapered to 5 mg daily.

**Figure 1.**
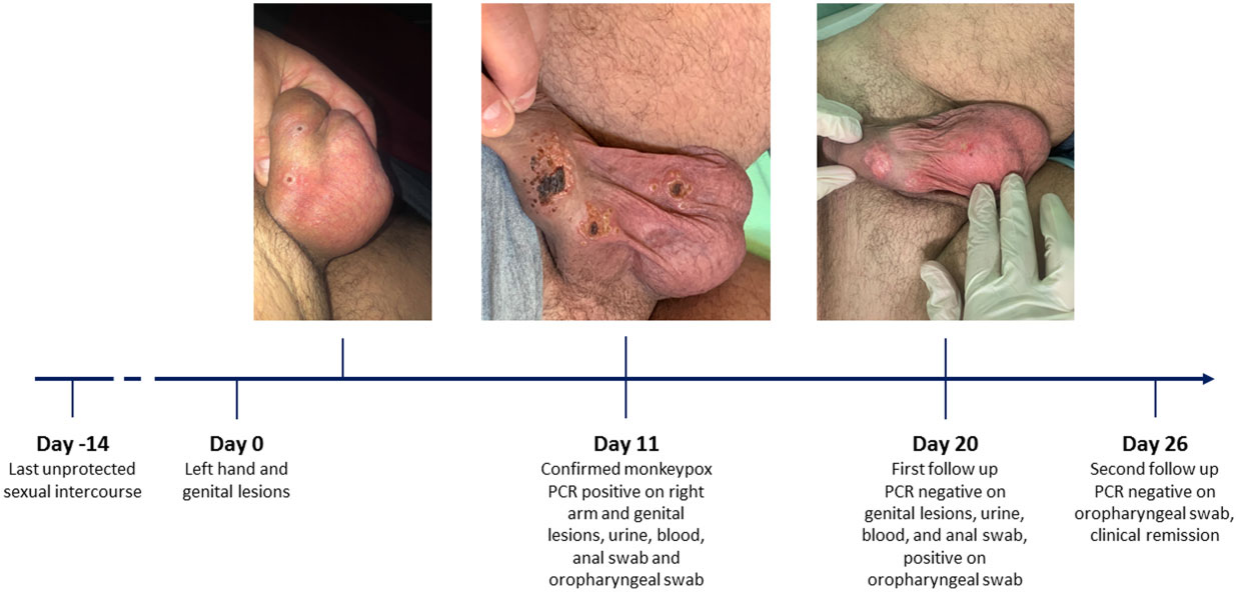
Clinical evolution of the patient until complete negativization and remission

The patient reported several unprotected sexual intercourses (the last 14 days before the skin eruption) and had been vaccinated for smallpox in childhood. At day 8 after the onset of the first signs, amoxicillin/clavulanate (875/125 mg three times daily for five days) was started because of enlargement and numerical increase of lesions. At day 11 he was evaluated at our sexually transmitted disease clinic, where we observed a necrotic evolution of lesions on left hand, penis and scrotum (Fig. 1), the appearance of new pustular lesions on both arms, chest, glutes, legs and feet, and mild systemic symptoms. Real-time polymerase chain reaction (rt-PCR) for non-variola orthopoxvirus DNA on genital and right arm lesions, anal and oropharyngeal swabs, plasma and urine resulted positive, with a positive confirmatory rt-PCR for MPXV DNA on right-arm lesion. Screening for sexually transmitted infections (including syphilis, HIV, chlamydia and Neisseria) resulted negative. Considering the rapid increase in the number of skin lesions and the still unknown evolution of MPX in fragile individuals, from day 12 to day 16 the patient was hospitalized. Given the good control of UCTD, the absence of data about the duration of MPX in immunosuppressed people, and the possible need of antiviral therapies, we decided to hold off the treatment with hydroxychloroquine and prednisone. We then observed a progressive clinical improvement and the patient did not need rescue therapy. Genital lesions, anal swab, urine and plasma became negative for MPXV at day 20, oropharyngeal swab at day 26, with clinical remission at day 26. Immunosuppressive therapy was then reintroduced. No flare of UCTD was observed throughout the entire period.

This is the first report of MPX in an individual with a rheumatologic disorder. In this case, the patient had undergone vaccination for smallpox, reported to be protective in 85% of individuals exposed to MPXV [8]. However, in this subject, we believe that possibly the use of steroid therapy and the prolonged time delay since vaccination might have played a role in its inefficacy.

Furthermore, skin lesions were compatible with classic MPX lesions, but scrotal and penile ones were greater in terms of dimensions and persistence and, globally, the eruption was rapidly worsening in the initial phase. It could be then speculated that a partially impaired antiviral response might have contributed to the patient’s disease features. It is unknown whether the prompt immunosuppression withdrawal might justify the positive outcome we observed since the patient’s admission.

Finally, time to clinical and virological remission in the case reported is at the upper limits of the previously described range of disease duration (2–4 weeks) [5], suggesting that immune landscape has an influence on disease persistence, even when immunosuppressive therapy is not aggressive.

MPXV infection is unfortunately rapidly spreading all over the world, especially in young male individuals. Although its manifestations seem mild to moderate, subjects with comorbidities, especially those who are on immunosuppressive therapy, such as rheumatic patients, might need to be strictly monitored in order to manage chronic therapy and prevent organ-threatening or even life-threatening complications.

## Data Availability

The data underlying this article will be shared on a reasonable request to the corresponding author.
